# Do Residents Believe that Onchocerciasis Transmission Was Eliminated? Results of A Post-Treatment Surveillance Knowledge, Attitude, and Practices Survey in Three Foci of Uganda

**DOI:** 10.4269/ajtmh.23-0906

**Published:** 2024-06-25

**Authors:** Lauri Bernard, Annet T. Khainza, Edson Byamukama, Moses Katabarwa, Jenna Coalson, Gregory S. Noland, Frank O. Richards

**Affiliations:** ^1^ River Blindness Elimination Program, The Carter Center, Atlanta, Georgia;; ^2^ River Blindness Elimination Program, The Carter Center, Kampala, Uganda

## Abstract

In Uganda, 15 of 17 foci have interrupted transmission of onchocerciasis (river blindness) and stopped mass drug administration (MDA) of ivermectin. This 2016 study describes the results of a knowledge, attitude, and practices survey regarding river blindness among participants (*N* = 1,577) 3–5 years after ivermectin MDA was halted in three foci: Imaramagambo halted in 2012, Kashoya-Kitomi in 2013, and Mt. Elgon in 2011. The study showed high levels of composite knowledge (focus-specific range: 66.8–81.2%) related to river blindness transmission, signs, symptoms, and treatment. However, 38.1% of respondents did not know that blackflies transmitted river blindness. Notably, 72.2% claimed they had not been informed why MDA was stopped, 56.3% did not believe river blindness had been eliminated, and 83.1% wanted ivermectin MDA to resume. During the 3–5 year post-treatment surveillance period, only 27.7% (438 of 1,577) reported being informed of what to do once treatments stopped, with the most knowledgeable hailing from the Mt. Elgon focus (47.9%). This study reinforces the need for programs to intensify health education and information dissemination when MDA is stopped. Programs must remind residents that although biting insects may persist, they no longer transmit river blindness. Incorporating messages about the elimination of river blindness into community health education campaigns can help improve the community’s perceptions related to the disease’s absence and the ending of a long-standing MDA intervention.

## INTRODUCTION

Onchocerciasis (river blindness [RB]) is a neglected tropical disease (NTD) caused by the nematode parasite *Onchocerca volvulus,* transmitted through the bites of infected female blackflies (*Simulium* species) that breed in fast-flowing rivers.^1^ Infection can cause skin damage, visual impairment, and in some cases, blindness if treatment is not administered.^2^ In Uganda, RB is transmitted by two vectors: *Simulium neavei* and *Simulium damnosum*.

River blindness was initially endemic in 17 transmission foci in 37 districts of Uganda. About 6.7 million persons were originally at risk in the country.^3^ Uganda launched control programs against RB in the 1950s using dichloro-diphenyl-trichloroethane (DDT) and successfully eliminated RB transmission in the Victoria focus in the 1970s.^4^ It later switched from vector control to annual community-directed mass drug administration (MDA) using ivermectin in 1987.^5^ In 2007, the government of Uganda transitioned its RB program from a strategy of morbidity control to one of transmission elimination based on a twice-per-year ivermectin MDA approach developed in the Americas^6^ plus a vector elimination strategy using temephos river dosing in foci where *S. neavei* was the vector. In areas where *S. damnosum* was the vector, targeted river dosing was done when feasible and necessary. In 2008, the Ugandan Ministry of Health established the Uganda Onchocerciasis Elimination Expert Advisory Committee (UOEEAC) to ensure that elimination decisions were grounded on the best scientific and technical counsel. In 2011, the UOEEAC established the national onchocerciasis elimination guidelines.^7^ Stopping MDA requires meeting WHO thresholds for transmission interruption using serological criteria (Ov16 antibody in children measured in Uganda by the “OEPA” ELISA)^8^ and entomological criteria (O-150 for evidence of the parasite in vectors using polymerase chain reaction).^9^ After MDA has been stopped, post-treatment surveillance (PTS) is recommended for 3–5 years to monitor for evidence of resumed transmission. Post-treatment surveillance includes vector monitoring to assess vector infection and health education to inform the communities why ivermectin treatments were stopped and how to sustain transmission interruption. As of 2023, Uganda had interrupted transmission and stopped MDA in 15 of the original 17 foci using these strategies.

Understanding what community residents know about RB and their beliefs is crucial. Assessing knowledge levels can help programs tailor health education efforts to improve understanding. Evaluating attitudes helps identify concerns that may hinder the acceptance of disease elimination and halting treatment. Assessing knowledge, attitude, and practices (KAP) may enhance the effectiveness of the RB elimination program. This article reports the results of a cross-sectional KAP survey in Uganda 3 or more years after MDA and vector control interventions were stopped in selected areas.

## MATERIALS AND METHODS

### Study area.

The study was conducted in three foci: Imaramagambo, Kashoya-Kitomi, and Mt. Elgon, which collectively had a population of 712,000 people. These foci were among the first in Uganda where RB transmission was successfully eliminated.^10^^–^^12^ Following recommendations from the UOEEAC between 2009 and 2013, the Ugandan Ministry of Health ceased ivermectin MDA. It began PTS in six foci: Kashoya-Kitomi, Imaramagambo, Itwara, Mpamba-Nkusi, Mt. Elgon, and Wambabya-Rwamarongo.

**Figure 1.**
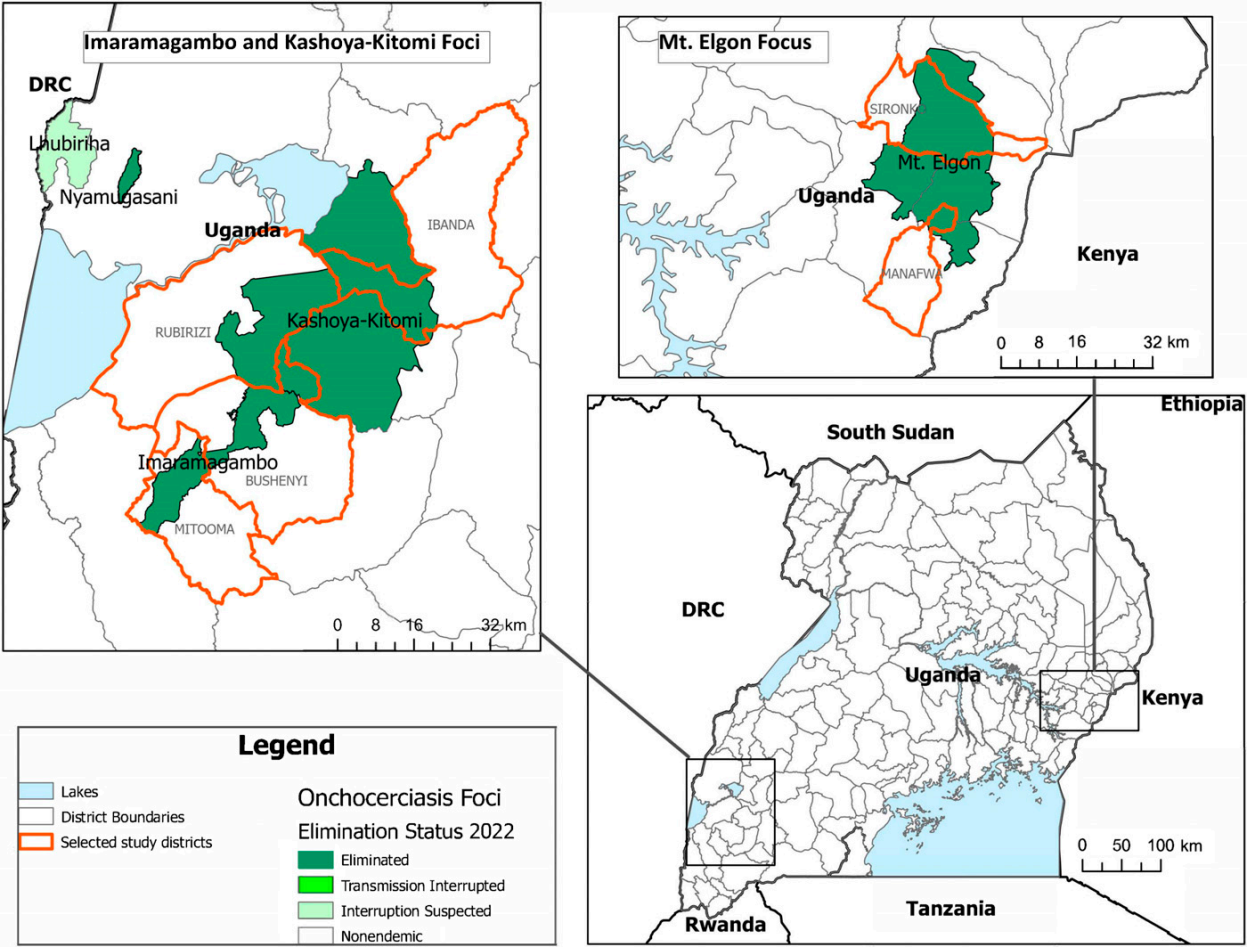
Map of selected study districts (red outline) and associated river blindness transmission foci. DRC = Democratic Republic of Congo.

For the KAP study, three of these six foci were randomly chosen: Imaramagambo (covering 1.66 km^2^ with a population of 116,000 people), Mt. Elgon (covering 693 km^2^ with a population of about 374,000), and Kashoya-Kitomi (covering 1,401 km^2^ with a population of 222,000) (Figure 1). Imaramagambo, comprising Bushenyi and Mitooma districts, had received 17 annual rounds of MDA over 20 years, successfully interrupting transmission in 2009. Mt. Elgon, encompassing Bududa, Manafwa, Mbale, and Sironko districts, underwent 23 rounds of MDA over 18 years, achieving transmission interruption in 2011. Kashoya-Kitomi (encompassing Buhweju, Kamwenge, Ibanda, and Rubirizi districts) underwent 25 rounds of MDA over 19 years and successfully halted transmission in 2013. In Mt. Elgon and Kashoya-Kitomi, vector elimination using temephos larvicide was also implemented in addition to biannual MDA.

### Sampling method.

A cross-sectional, multistage, random survey was conducted in the three selected foci. In Imaramagambo, both districts were selected; in Mt. Elgon and Kashoya-Kitomi, two districts in each focus were chosen randomly. Lower administrative units (sub-counties, parishes, and communities) were selected randomly based on their respective lists. Within districts, the selection process resulted in selecting at least 30% of the units at each administrative level. The number of randomly chosen communities was determined proportionally based on the communities and populations in each district. A list of households was generated in each selected community from the household register used during ivermectin MDA. The number of households was divided by the required number to find the skip interval. Using a random start point on the list and then the regular skip interval for subsequent households, 15–20 households were systematically selected per community, with one individual (typically the head of household) selected for face-to-face interviews. The study area was estimated to have about 160,310 households. Using Salant and Dillman’s formula^13^ with a 95% CI and a margin of error of 0.025 to quantify the proportion of household representatives holding each knowledge and belief outcome categorized as a binary variable, conservatively assuming 50%, the target sample size was 1,522 households. To account for a nonresponse rate of up to 5%, the final targeted sample size was 1,602 households.

In each selected household, one interview was conducted with the head of the family. Sex was chosen using an alternating sampling pattern. The eldest child (>18 years) was interviewed if the household heads were absent. If the selected sex was absent, the opposite sex was questioned. Polygamous families were included as separate households. The husband was interviewed as the head of one household, whereas the wives were interviewed as the heads of additional households, each wife representing one household. If a household was nonresponsive, such as being absent, it was not substituted with another household. Similarly, households whose religious beliefs prohibited participation or in which the individual was ill, deaf, or mute were not substituted. To prevent bias, 926 individuals who previously worked as community drug distributors and community supervisors for the Community-Directed Treatment with Ivermectin (CDTI) program were not included as interviewers or subjects for this study.

### Questionnaires.

The survey tool was a standardized questionnaire adapted from a Guatemala PTS survey^14^ conducted by the senior author (F. O. Richards) and adjusted to suit the local Ugandan context. The research team reviewed the semistructured questionnaire to ensure that the questions were clear and understandable before application in the three selected foci. The questionnaire included the following components: 1) background information: sex, age, marital status, gainful activities the respondent was involved in [not reported], and their length of residency in the community; 2) knowledge of the disease: Yes/No questions including if the respondent knew of RB and the symptoms, had ever seen someone in the community with the disease/symptoms, had ever been treated for the disease, and knew of others in the community treated for the disease, how a person contracts RB, and if there were other ways of controlling the disease (e.g., vector control); and 3) elimination: had the respondent been told why ivermectin was being stopped, had the disease been eliminated, and should treatment with ivermectin (Mectizan or the “RB medicine”) be continued?

### Interviewer selection and training.

Interviews were conducted by trained villagers who received a modest stipend in compensation for their time. District Onchocerciasis Coordinators assisted in identifying two interviewers from each village for training. They also assisted in the mobilization of the lower-level participants and selected the training venue. Interviewers were required to be fluent in the local language and to read and write English. The interviewers were taught skip patterns, technical terminology, and singular and multiple response situations in the questionnaires. They were instructed to ask questions conversationally. The principal investigator and research supervisors trained the interviewers for 1 day. They were expected to complete their interviews (an average of 7.5 per community) in 5 days. Responses were captured on paper forms and reviewed by the research team. If contradictions were noticed, the team revisited the household for clarification.

### Ethical approval.

The protocol for this study was deemed nonresearch (routine program activity) by the Emory University Institutional Review Board and the Uganda National Council for Science and Technology. Written and oral informed consent was obtained from community leaders and selected household heads before beginning the survey. Community members were told that the results would improve program activities targeted for RB-endemic areas receiving interventions and areas where interventions were halted. There were no repercussions for individuals who opted out of the study.

## STATISTICAL ANALYSES

Survey data were coded and entered in Epi Info 7^15^ (U.S. Centers for Disease Control and Prevention, Atlanta, GA). A composite binary knowledge indicator was created based on respondents’ scores across the four general knowledge questions: “knowledgeable” if they correctly answered at least two of the general knowledge questions and “not knowledgeable” if they correctly answered 1 or none. Descriptive, unweighted statistics were reported for this cross-sectional survey, and χ^2^ tests were used to test for associations between independent factors and outcomes. Logistic regression was used to test for the strength of associations between variables. Variables were considered statistically significant at *P* <0.05.

## RESULTS

The surveys were conducted between May and July 2016 in the three foci, and 1,577 interviews were completed in 87 communities (Table 1). Surveys were relatively evenly distributed across the foci, with 36.4% conducted in Mt. Elgon, 34.4% in Imaramagambo, and 29.2% in Kashoya-Kitomi. The nonresponse rate was 5%. Most household heads who were not interviewed had temporarily migrated to other areas for employment and farming opportunities.

**Table 1:** Social demographics/characteristics of survey participants

District	Overall, *N* (%)	Imaramagambo, *n* (%)	Kashoya-Kitomi, *n* (%)	Mt. Elgon, *n* (%)	*P*-Value*
Communities Selected	87	28	26	33	–
Household Representatives	1,577	542 (34.4)	461 (29.2)	574 (36.4)	–
Sex					
Male	796 (50.5)	260 (48.0)	237 (51.4)	299 (52.1)	0.346
Female	781 (49.5)	282 (52.0)	224 (48.6)	275 (47.9)	
Marital Status					
Married	1,216 (77.1)	418 (77.1)	376 (81.6)	422 (73.5)	<0.001
Single	147 (9.3)	39 (7.2)	18 (3.9)	90 (15.7)	
Widow/Widower	214 (13.6)	85 (15.7)	67 (14.5)	66 (10.8)	
Median Age (IQR) (years)	41 (24)	40 (22)	40 (20)	43 (27)	
Age (years)					
<30	349 (22.1)	114 (21.0)	110 (23.9)	125 (21.8)	0.002
30–49	726 (46.0)	271 (50.0)	223 (48.4)	232 (40.4)	
50+	502 (31.8)	157 (29.0)	128 (27.8)	217 (37.8)	
Years in Community					
<2	41 (2.6)	20 (3.7)	15 (3.3)	6 (1.0)	<0.001
2–5	95 (6.0)	39 (7.2)	31 (6.7)	25 (4.4)	
6–10	154 (9.8)	74 (13.7)	48 (10.4)	32 (5.6)	
>10	1,286 (81.6)	409 (75.5)	366 (79.6)	511 (89.0)	

IQR = interquartile range.

* *P*-value based on χ^2^ tests of association.

### Social demographics/characteristics of survey participants.

Sex distribution among the participants was nearly equal, with 50.5% male and 49.5% female (Table 1). Marital status and age demographics revealed that most participants (77.1%) were married. The median age of survey participants across the three foci was 41 years, with an interquartile range of 24 years. The age of respondents was significantly higher in Mt. Elgon (median: 43 years) than in Imaramagambo (40 years) and Kashoya-Kitomi (40 years). In addition, most participants (81.6%) had resided in their respective communities for over a decade. Across the districts, crop farming was the main occupation held.

### Knowledge of RB by focus.

Overall, the study’s participants exhibited a high knowledge of RB. Most respondents (91.3% overall) indicated they were aware of RB, and 70.3% had seen people with signs and symptoms of the disease (Table 2). Fewer people (61.9%) correctly knew how a person gets the disease, but a higher proportion (77.9%) knew where a person could get it. Except for knowing a disease called RB, variations in knowledge responses were statistically significant between the three foci. Composite RB knowledge, derived from answers to the preceding questions, was high overall (73.5%) but significantly differed across foci (from 66.8% in Imaramagambo to 81.8% in Mt. Elgon).

**Table 2:** Knowledge of river blindness by focus

Variable	Overall (*N* = 1,577), *N* (%)	Focus			*P*-Value*
		Imaramagambo, *n* (%)	Kashoya-Kitomi, *n* (%)	Mt. Elgon, *n* (%)	
Know a Disease Called RB					
No	135 (8.6)	43 (7.9)	36 (7.8)	56 (9.8)	0.444
Yes	1,440 (91.3)	498 (92.1)	424 (92.2)	518 (90.2)	
Ever Seen People With RB Signs and Symptoms^†^					
No	428 (29.7)	143 (28.8)	81 (19.1)	204 (39.4)	<0.001
Yes	1,012 (70.3)	354 (71.2)	344 (80.9)	314 (60.6)	
Know How a Person Gets RB					
No	600 (38.1)	248 (45.8)	259 (56.2)	93 (16.2)	<0.001
Yes	977 (61.9)	294 (54.2)	202 (43.8)	481 (83.8)	
Knows Which Places People Can Get RB					
No	347 (22.1)	163 (30.0)	86 (18.7)	98 (17.1)	<0.001
Yes	1,224 (77.9)	375 (69.7)	374 (81.3)	475 (82.9)	
Overall Knowledge Status^‡^					
Not Knowledgeable	418 (26.5)	180 (33.2)	130 (28.2)	108 (18.8)	<0.001
Knowledgeable	1,159 (73.5)	362 (66.8)	331 (71.8)	466 (81.2)	
Total	1,577	542	461	574	

RB = river blindness.

* *P*-value based on a χ^2^ test of association.

^†^ Question was asked only of the 1,440 participants who said they knew a disease called RB.

^‡^ Respondents were classified as knowledgeable if they knew at least two of the four questions listed above and not knowledgeable if they knew only one or none.

### Knowledge about RB by demographic characteristics.

Table 3 explores associations between the composite RB knowledge indicator and various demographic factors. Participants in the Mt. Elgon focus exhibited 2.1 higher odds of disease knowledge than those from Imaramagambo (*P* <0.001). Sex-based differences in knowledge level were apparent, as men showed 1.7 times higher odds of being knowledgeable than women (*P* <0.001). Age-related trends in knowledge were evident, showing that respondents aged 30–49 years and those over 50 years had 2.2 and 2.8 higher odds, respectively, of being informed about RB compared with those under 30 years old (*P* <0.001). Length of residency in the community was also associated with knowledge about RB: Participants who had resided in their communities for more than a decade (10 years) were found to have 8.9 times higher odds of being knowledgeable about the disease compared with those who lived in communities for less than 2 years (*P* <0.001).

**Table 3:** Knowledge of river blindness by demographic characteristics

Variable	Total (*N* = 1,577), *N* (%)	Overall RB Knowledge Status*		χ^2^ *P*-Value^†^	OR^‡^ (95% CI)	*P*-Value^‡^
		Not Knowledgeable (*N* = 418), *n* (%)	Knowledgeable (*N* = 1,159), *n* (%)			
Focus						
Imaramagambo	542 (34.4)	180 (33.2)	362 (66.8)	<0.001	1	
Kashoya-Kitomi	461 (29.2)	130 (28.2)	331 (71.8)		1.3 (0.9, 1.7)	0.087
Mt. Elgon	574 (36.4)	108 (18.8)	466 (81.2)		2.1 (1.6, 2.8)	<0.001
District						
Bushenyi/Imaramagambo	254 (16.1)	85 (33.5)	169 (66.5)	<0.001	1	–
Mitooma/Imaramagambo	288 (18.3)	95 (33.0)	193 (67.0)		1.0 (0.7, 1.5)	0.906
Ibanda/Kashoya-Kitomi	119 (7.5)	14 (11.8)	105 (88.2)		3.8 (2.0, 7.0)	<0.001
Rubirizi/Kashoya-Kitomi	342 (21.7)	116 (33.9)	226 (66.1)		0.9 (0.6, 1.4)	0.908
Manafwa/Mt. Elgon	203 (12.9)	48 (23.6)	155 (76.4)		1.6 (1.1, 2.5)	0.022
Sironko/Mt. Elgon	371 (23.5)	60 (16.2)	311 (83.8)		2.6 (1.8, 3.8)	<0.001
Sex						
Male	796 (50.5)	172 (21.6)	624 (78.4)	<0.001	1.7 (1.3, 2.1)	<0.001
Female	781 (49.5)	246 (31.5)	535 (68.5)		1	–
Marital Status						
Married	1,216 (77.1)	321 (26.4)	895 (73.6)	0.955	1	–
Single	147 (9.3)	39 (26.5)	108 (72.5)		0.9 (0.7,1.5)	0.973
Widow	164 (10.4)	43 (26.2)	121 (73.8)		1.0 (0.6, 1.5)	0.961
Widower	50 (3.2)	15 (30.0)	35 (70.0)		0.8 (0.5, 1.6)	0.572
Age (years)						
<30	349 (22.1)	143 (41.0)	206 (59.0)	<0.001	1	–
30–49	726 (46.0)	175 (24.1)	551 (75.9)		2.2 (1.7, 2.9)	<0.001
50+	502 (31.8)	100 (19.9)	402 (80.1)		2.8 (2.0, 3.8)	<0.001
Years in Community						
<2	41 (2.6)	29 (70.7)	12 (29.3)	<0.001	1	–
2–5	95 (6.0)	48 (50.5)	47 (49.5)		2.4 (1.1, 5.2)	0.031
6–10	154 (9.8)	65 (42.2)	89 (57.8)		3.3 (1.6, 7.0)	0.002
>10	1,286 (81.6)	275 (21.4)	1,011 (78.6)		8.9 (4.5, 17.6)	<0.001

OR = odds ratio; RB = river blindness.

* Respondents were classified as knowledgeable about RB if they knew at least two of the four questions in Table 2 and not knowledgeable if they knew only one or none.

^†^ *P*-value based on χ^2^ test of association.

^‡^ OR based on simple logistic regression.

### River blindness elimination knowledge by focus.

Table 4 presents the analysis of community knowledge regarding RB elimination across the three foci. The combined findings revealed that more than 85.7% of the participants reported being unaware of anyone in the community currently suffering from the disease. This was similar for all the foci. Most (85.5%) reported witnessing people ever being treated in their communities. Approximately 83.7% of respondents reported that they were treated with ivermectin. However, a minority of respondents said they had been informed of what to do when ivermectin stopped, ranging from only 7.4% in Kashoya-Kitomi to 23.8% in Imaramagambo to 47.9% in Mt. Elgon (*P* <0.001). Overall, only 14.1% of respondents knew that other interventions besides ivermectin treatment, such as vector control, were used to eliminate the disease. Awareness of other interventions was low even in Kashoya-Kitomi (7.8%) and Mt. Elgon (16.7%), where the program applied vector control in addition to ivermectin MDA. Less than half of the participants (43.7%) believed that RB had been eliminated, and a large majority (83.1%) wanted the ivermectin treatment program to continue. Mt. Elgon, the best-informed focus by composite knowledge indicator, had the lowest proportion (70.4%) of respondents wanting ivermectin MDA to continue (compared with 87.6% and 93.7% in Imaramagambo and Kashoya-Kitomi, respectively) and the highest percentage of respondents (68.1%) who believed RB had been eliminated (compared with 36.7% in Imaramagambo and 21.7% in Kashoya-Kitomi). Differences across foci were statistically significant for most of these factors.

**Table 4:** River blindness elimination knowledge by transmission focus

Variable	Total (*N* = 1,577), *N* (%)	Imaramagambo (*N* = 542), *n* (%)	Kashoya-Kitomi (*N* = 461), *n* (%)	Mt. Elgon (*N* = 574), *n* (%)	*P*-Value*
Aware of Anyone in the Community Who Is Currently Suffering from RB					
Yes	226 (14.3)	73 (13.5)	65 (14.1)	88 (15.3)	0.665
No	1,351 (85.7)	469 (86.5)	396 (85.9)	486 (84.7)	
People Ever Been Treated in the Community					
Yes	1,349 (85.5)	448 (82.7)	403 (87.4)	498 (86.8)	0.059
No	228 (14.5)	94 (17.3)	58 (12.6)	76 (13.2)	
Ever Been Treated					
Yes	1,320 (83.7)	412 (76.0)	405 (87.9)	503 (87.6)	<0.001
No	257 (16.3)	130 (24.0)	56 (12.1)	71 (12.4)	
Informed What to Do Next When Treatment of RB Was Stopped					
Yes	438 (27.8)	129 (23.8)	34 (7.4)	275 (47.9)	<0.001
No	1,139 (72.2)	413 (76.2)	427 (92.6)	299 (52.1)	
Any Other Interventions Used to Fight RB					
Yes	222 (14.1)	90 (16.6)	36 (7.8)	96 (16.7)	<0.001
No	1,355 (85.9)	452 (83.4)	425 (92.2)	478 (83.3)	
Was RB Eliminated					
Yes	689 (43.7)	198 (36.5)	100 (21.7)	391 (68.1)	<0.001
No	888 (56.3)	344 (63.5)	361 (78.3)	183 (31.9)	
Should Treatment Continue					
Yes	1,311 (83.1)	475 (87.6)	432 (93.7)	404 (70.4)	<0.001
No	266 (16.9)	67 (12.4)	29 (6.3)	170 (29.6)	

RB = river blindness.

* *P*-value based on χ^2^ test of association.

### Comparison of respondents’ belief that RB was eliminated and demographic characteristics.

Table 5 analyzes the relationship between the belief in RB elimination and various demographic factors. Within foci, significant differences were evident between districts (*P* <0.001), with a majority of those in the Mt. Elgon region (68.1%) believing that RB had been eliminated. Sex-based differences were not significant. When considering marital status and viewpoints, unmarried respondents (53.7%) were likelier to believe that the disease had been eliminated, whereas only 43.9% of married participants shared this perspective. However, only 35.5% of widowed participants believed that RB was eliminated. The impact of age on believing in elimination was small, and there was no significant difference in belief across diverse age groups. The length of residency did influence the perception that the disease had been eliminated. Overall, 43.7% of the participants believed that RB was eliminated; this percentage dropped to only 17.1% among those who had lived in the community for less than 2 years.

**Table 5:** Belief in river blindness elimination by demographic characteristics

Variable	Total (*N* = 1,577), *N* (%)	Believe That RB Was Eliminated		*P*-Value*
		No (*N* = 888), *n* (%)	Yes (*N* = 689), *n* (%)	
Focus				
Imaramagambo	542 (34.4)	344 (63.5)	198 (36.5)	<0.001
Kashoya-Kitomi	461 (29.2)	361 (78.3)	100 (21.7)	
Mt. Elgon	574 (36.4)	183 (31.9)	391 (68.1)	
District				
Bushenyi/Imaramagambo	254 (16.1)	171 (67.3)	83 (32.7)	<0.001
Mitooma/Imaramagambo	288 (18.3)	173 (60.1)	115 (39.9)	
Ibanda/Kashoya-Kitomi	119 (7.5)	109 (91.6)	10 (8.4)	
Rubirizi/Kashoya-Kitomi	342 (21.7)	252 (73.7)	90 (26.3)	
Manafwa/Mt. Elgon	203 (12.9)	85 (41.9)	118 (58.1)	
Sironko/Mt. Elgon	371 (23.5)	98 (26.4)	273 (73.6)	
Sex				
Male	796 (50.5)	435 (54.6)	361 (45.4)	0.179
Female	781 (49.5)	453 (58.0)	328 (42.0)	
Marital Status				
Married	1,216 (77.1)	682 (56.1)	534 (43.9)	0.003
Single	147 (9.3)	68 (46.3)	79 (53.7)	
Widow/Widower	214 (13.6)	138 (64.5)	76 (35.5)	
Participant’s Age (years)				
<30	349 (22.1)	199 (57.0)	150 (43.0)	0.823
30–49	726 (46.0)	412 (56.7)	314 (43.3)	
50+	502 (31.8)	277 (55.2)	225 (44.8)	
Years in Community				
<2	41 (2.6)	34 (82.9)	7 (17.1)	0.003
2–5	95 (6.0)	54 (56.8)	41 (43.2)	
6–10	154 (9.8)	93 (60.4)	61 (39.6)	
>10	1,286 (81.6)	706 (54.9)	580 (45.1)	

RB = river blindness.

* *P*-value based on χ^2^ test of association.

### Community belief and attitude toward RB elimination.

Table 6 examines the community’s perspective on RB elimination, providing valuable insight into their beliefs and attitudes. The findings showed that of the participants who believed RB was eliminated (43.7%), 35.8% believed individuals in their community were still affected by it (*P* <0.001). Among the 85.5% who knew of others treated, only 46.5% believed that RB had been eliminated. Of the participants treated for RB (83.7%), 46.6% thought the disease had been successfully eliminated.

**Table 6:** Belief in RB elimination compared with other RB knowledge and attitudes

Variable	Total, *N* (%)	Believe That RB Was Eliminated		*P*-Value*
		No, *n* (%)	Yes, *n* (%)	
Aware of Anyone in the Community Who Is Currently Suffering from RB (*N* = 1,438)^†^				
Yes	226 (15.7)	145 (64.2)	81 (35.8)	<0.001
No	1,212 (84.3)	634 (52.3)	578 (47.7)	
People Ever Been Treated in the Community (*N* = 1,440)^†^				
Yes	1,349 (93.7)	722 (53.5)	627 (46.5)	0.166
No	91 (6.4)	58 (63.7)	33 (36.3)	
Ever Been Treated (*N* = 1,438)^†^				
Yes	1,320 (91.8)	705 (53.4)	615 (46.6)	0.034
No	118 (8.2)	75 (63.6)	43 (36.4)	
Informed What to Do Next When Treatment of RB Was Stopped (*N* = 1,573)				
Yes	438 (27.8)	86 (19.6)	352 (80.3)	<0.001
No	1,135 (72.2)	798 (70.3)	337 (29.7)	
Any Other Interventions Used to Fight RB (*N* = 1,572)				
Yes	222 (14.1)	84 (37.8)	138 (62.2)	<0.001
No	1,350 (85.9)	799 (59.2)	551 (40.8)	
Should Treatment with Ivermectin Continue (*N* = 1,574)				
Yes	1,311 (83.3)	836 (63.8)	475 (36.2)	<0.001
No	263 (16.7)	49 (18.6)	214 (81.3)	

RB = river blindness.

* *P*-value based on χ^2^ test of association.

† This question was asked only to the 1,440 participants who stated they “knew disease called RB.”

However, among 27.8% of participants who were informed about post-MDA procedures, a significant majority (80.3%) continued to believe in the elimination of RB from their community (*P* <0.001). Only 14.1% were aware of the alternative strategy used for RB elimination. Among this subgroup, 62.2% believe it was eliminated, a belief significantly associated with knowledge of RB elimination (*P* <0.001).

Most importantly, a substantial majority (83.1%) advocated continuing ivermectin. Within this group, 36.2% believed that RB elimination had occurred.

## DISCUSSION

The findings of this study show variable KAP between the three foci, where ivermectin MDA had been stopped after WHO thresholds to declare elimination had been met. After decades of annual and semiannual MDA, it is not surprising that 91.3% of the overall study participants were knowledgeable about RB. This high degree of knowledge aligns with a KAP study conducted in four communities within Gesha town, southwest Ethiopia, where 89.4% of the community demonstrated awareness of the disease.^16^ This could be attributed to the health education and disease awareness campaigns conducted during the intervention phase when high-coverage rates necessary for interrupting transmission were being established.

On average, 70% of participants reported witnessing others afflicted by the disease. Across the three foci, the Kashoya-Kitomi focus exhibited the highest percentage (80.9%) of individuals encountering such cases, whereas Mt. Elgon had the lowest rate (60.6%). This disparity may stem from the initially high prevalence or endemicity of Kashoya-Kitomi focus during the early stages of treatment. Moreover, it is possible that some participants were affected by the disease.

Those who had resided in the communities longer were likely to be more knowledgeable about RB than those who had lived there for fewer years. This is probably because of their interaction with the program for more extended periods than those new to the area, who may not have had experience with the MDA program.

The most knowledgeable KAP results came from the Mt. Elgon focus interviews. Respondents of the Mt. Elgon focus had a higher percentage (47.9%) of knowledge about the next steps after stopping MDA and a higher percentage (68.1%) of those who believed RB was eliminated. Mt. Elgon also had the lowest proportion (70.4%) of respondents wanting ivermectin MDA to continue. The data indicated that foci that had better information about the decision to stop MDA would have led to better elimination acceptance and less desire to reinstate MDA. A minority of respondents in Kashoya-Kitomi (7.4%) and Imaramagambo (23.8%) said they had been informed of what to do when ivermectin stopped, whereas Mt. Elgon respondents were better informed. This can be attributed to more effective health education campaigns conducted in Mt. Elgon before and during PTS. Mt. Elgon’s results are also consistent with those of the Guatemalan KAP study, indicating that a thorough comprehension of disease elimination is directly linked to understanding of the rationale for stopping MDA.^14^ This parallels the approach observed in the elimination strategy for Guinea worm disease, where robust health education initiatives significantly contributed to participants’ knowledge regarding disease eradication.^17^ This highlights the importance of sustained community dialogue and ongoing health education to monitor NTDs earmarked for elimination, particularly where MDA is being halted (such as onchocerciasis, lymphatic filariasis, and trachoma). National programs could enhance their efforts by conducting comprehensive and scripted health education campaigns on halting MDA, which would continue regularly until the end of the PTS period. In Uganda, the health education intervention for discontinuing MDA lacked standardization and scripting and varied by focus. Perhaps as a result, fewer than half of the participants believed that RB elimination had been achieved.

To eliminate the disease, Uganda adopted the strategies of biannual treatment with ivermectin and vector elimination or targeted control, where feasible, through larviciding.^7^ Interestingly, only 14.1% of participants knew about other interventions in addition to ivermectin used to achieve elimination. However, larviciding was usually applied inside thick forests and on selected sites along the rivers at intervals measured in kilometers and determined by larvicide carriage distance. The dosing sites were usually far from community settlements, so most community members never witnessed the river dosing exercise. Only community leaders and trained community members who assisted the entomological teams saw vector elimination exercises. Larviciding local rivers was an essential element of the elimination program in Kashoya-Kitomi and Mt. Elgon. In Imaramagambo, the vector might have vanished because agricultural chemicals used in the large tea plantations may have affected their breeding sites.

When looking at participants’ KAP by sex, male participants were more knowledgeable about RB disease elimination than women. Interestingly, participants who believed the disease was eliminated exhibited a higher level of understanding, with 81% comprehending disease transmission compared with only 47% among those who did not believe the disease was eliminated. This implies that enhancing knowledge through health education messaging could clarify the Ministry of Health’s rationale for discontinuing the ivermectin MDA program.

A significant finding of this study was that the vast majority (83.1%) of participants across the three foci wanted ivermectin distribution to continue in their respective areas, even among those who believed RB had been eliminated (68.9% of whom wanted MDA to continue) compared with those who did not believe in elimination (94.4% who favored MDA continuing). This finding could be attributed to the other therapeutic benefits of ivermectin in preventing intestinal worms or ectoparasites such as scabies, as was the case in the Guatemala study.^14^ It could also be attributed to the participants’ need for more knowledge regarding RB elimination. Communities can be expected to continue to want ivermectin either because they do not believe RB has been eliminated or because they desire the other beneficial effects of ivermectin despite the disease’s absence. This confirms the importance of informing communities about the plan to stop ivermectin treatment and understanding why they may react negatively to this news.

In 2014, Richards et al.^14^ conducted a post-MDA KAP survey of 148 interviews with heads of households in four formerly hyperendemic communities in Guatemala. The survey was conducted 3 years after MDA was stopped, and questions similar to those in the Uganda study were posed. Almost all the Guatemalan respondents had had onchocerciasis and had taken ivermectin. Older respondents were more likely to have had the infection or have known someone afflicted by it. Knowledge of transmission could have been better, with only 44% knowing an insect transmitted onchocerciasis. In contrast to the findings reported in this study from Uganda, in Guatemala, the majority (69%) of respondents had been informed by an intensive Ministry of Health Education campaign, which coincided with the conclusion of the MDA program. Despite that information campaign, the numbers in Guatemala in 2014 were very similar to those in Uganda in 2020: Half of all Guatemalan respondents were skeptical that elimination had occurred, and 60% wanted the ivermectin MDA program to continue. Based on this experience, one might argue that ivermectin’s popularity (in Guatemala’s case, where the effects on lice and intestinal worms were well recognized in survey questions) superseded any elimination messages delivered in a well-resourced stop-MDA informational program.

A limitation of this study is the absence of an analysis examining potential reasons why the community might still seek ivermectin delivery after being told that onchocerciasis had been eliminated. Based on the Guatemala experience, we suspect this could be due to the presence of biting insects or other conditions, such as scabies, and intestinal worms. For instance, a resurgence of scabies within these communities during the PTS period may have influenced the typical inclination toward continued treatment. A comprehensive understanding of the community’s motivations for renewing the ivermectin MDA program is essential but can only be addressed by conducting additional studies. Another limitation of the study was the omission of inquiries about respondents’ educational status. One notable strength of the study is the adoption of a non-replacement approach to sampling. Participants who were absent after a second follow-up were not substituted. Although non-replacement sampling may lead to a smaller final sample size, it can enhance the rigor and integrity of the study’s findings by reducing bias and maintaining consistency throughout the research process. Another strength is that the study did not include (as interviewers or respondents) the community drug distributors or supervisors who were directly involved in the CDTI program. Their inclusion might have introduced biased reporting, given their direct involvement in all MDA activities.

A more in-depth understanding of the KAP of the communities is essential for the future development of health education approaches in the immediate post-MDA era. Communities can be expected to continue to want ivermectin either because they do not believe onchocerciasis has been eliminated or because they desire the other beneficial effects that still come from treatment in the absence of onchocerciasis transmission. This means that further social science research and innovative communication methods are urgently needed to effectively approach the thousands of communities where ivermectin MDA is being withdrawn to fully engage them and meet them “where they are.”

Insufficient knowledge of RB elimination may stem from a lack of adequate integration of post-MDA messages within the health education packages encompassing other NTDs in integrated healthcare delivery systems. To prevent the recurrence of the disease, it is also essential to teach community residents how to do their part in passive post-elimination surveillance. For example, they could be made aware of what to do if they see someone with symptoms of onchocerciasis. The Ministry of Health must focus on its post-treatment and post-elimination surveillance responsibilities and follow WHO onchocerciasis elimination guidelines to ensure that health education training is being passed down from former community drug distributors and ask that they continue to volunteer to play an educational role in their communities.

## CONCLUSION

This study described residents’ KAP toward RB and ivermectin use in three post-endemic foci where ivermectin MDA had been stopped. Although there were substantial differences between the KAP in the three foci, most respondents said they had not been informed why MDA had been stopped, did not believe RB had been eliminated, and wanted MDA to resume. This underscores the significance of transparently communicating the intention to discontinue ivermectin treatment to communities. Effective communication is crucial for managing expectations, fostering trust, mitigating misinformation, promoting compliance, empowering residents, and facilitating their preparation for the post-MDA transition.

Determining community members’ KAP about external decisions that impact their health is an important feedback mechanism for health authorities. On simple ethical grounds, withdrawing a program that has been implemented at the community level for decades ought to be accompanied by an explanation by health authorities. Halting MDA should involve tested and effective communication that empowers communities to comprehend the rationale behind medication withdrawal and to accept that decision. This aspect remains largely unexplored in existing literature, with the exception of findings from Guatemala. Considerable resources are devoted to health education on initiating MDA to promote treatment-seeking behaviors to achieve high treatment coverage. However, this is not the case with stopping MDA, which does not have the same essential community “compliance” for success requirements.

The study findings reinforce the need for programs to intensify and standardize health education and information dissemination when MDA is stopped and during the 3–5-year PTS period. Key messages should include reminding residents that although biting insects are still present in the area, they no longer transmit the infection because of the success of past MDA treatment. The information presented in this study can be valuable to others for designing targeted awareness and education programs to improve a community’s knowledge and understanding of RB elimination.

## References

[1] Richards FO Miri E Meredith S Guderian R Sauerbrey M Remme H Packard R Ndiaye JM, 1998. Onchocerciasis. Bull World Health Organ 76 *(Suppl 2):* 147–149. 10063699 PMC2305701

[2] Katabarwa MN Eyamba A Nwane P Enyong P Yaya S Baldiagaï J Madi TK Yougouda A Andze GO Richards FO, 2011. Seventeen years of annual distribution of ivermectin has not interrupted onchocerciasis transmission in North Region, Cameroon. Am J Trop Med Hyg 85: 1041–1049. 22144441 10.4269/ajtmh.2011.11-0333 PMC3225149

[3] Katabarwa MN , 2018. After 70 years of fighting an age-old scourge, onchocerciasis in Uganda, the end is in sight. Int Health 10 *(Suppl_1):* i79–i88. 29471335 10.1093/inthealth/ihx044

[4] Katabarwa MN , 2020. Historical elimination of onchocerciasis from Victoria Nile Focus in Central Uganda verified using WHO criteria. Am J Trop Med Hyg 102: 1411–1416. 32228786 10.4269/ajtmh.20-0064 PMC7253126

[5] Sturchio JL, 2001. The case of ivermectin: Lessons and implications for improving access to care and treatment in developing countries. Community Eye Health 14: 22–23. 17491909 PMC1705916

[6] Katabarwa MN , 2012. Transmission of onchocerciasis in Wadelai focus of northwestern Uganda has been interrupted and the disease eliminated. J Parasitol Res 2012: 1–7. 10.1155/2012/748540 PMC3433138 22970347

[7] Uganda Ministry of Health , 2011. *Guidelines for Certification of Onchocerciasis Elimination in Uganda*. Available at: https://www.cartercenter.org/resources/pdfs/news/health_publications/river_blindness/Guidelines_for_Certification_of_Onchocerciasis_elimination_uganda.pdf. Accessed September 9, 2023.

[8] Richards FO , 2018. Operational performance of the *Onchocerca volvulus* “OEPA” Ov16 ELISA serological assay in mapping, guiding decisions to stop mass drug administration, and posttreatment surveillance surveys. Am J Trop Med Hyg 99: 749–752. 30014821 10.4269/ajtmh.18-0341 PMC6169192

[9] Higazi TB , 2011. Polymerase chain reaction pool screening used to compare prevalence of infective black flies in two onchocerciasis foci in Northern Sudan. Am J Trop Med Hyg 84: 753–756. 21540385 10.4269/ajtmh.2011.11-0009 PMC3083743

[10] Katabarwa MN , 2016. The Imaramagambo onchocerciasis focus in southwestern Uganda: Interruption of transmission after disappearance of the vector *Simulium neavei* and its associated freshwater crabs. Am J Trop Med Hyg 95: 417–425. 27215297 10.4269/ajtmh.16-0181 PMC4973193

[11] Katabarwa M , 2014. Transmission of *Onchocerca volvulus* by *Simulium neavei* in Mount Elgon focus of eastern Uganda has been interrupted. Am J Trop Med Hyg 90: 1159–1166. 24686740 10.4269/ajtmh.13-0501 PMC4047747

[12] Lakwo T , 2017. Interruption of the transmission of *Onchocerca volvulus* in the Kashoya-Kitomi focus, western Uganda by long-term ivermectin treatment and elimination of the vector *Simulium neavei* by larviciding. Acta Trop 167: 128–136. 28034767 10.1016/j.actatropica.2016.12.029

[13] Salant P Dillman DA, 1994. How to Conduct Your Own Survey. New York, NY: Wiley.

[14] Richards FO , 2016. A knowledge, attitudes and practices survey conducted three years after halting ivermectin mass treatment for onchocerciasis in Guatemala. PLoS Negl Trop Dis 10: e0004777. 27341104 10.1371/journal.pntd.0004777 PMC4920414

[15] Dean AG Dean JA Burton AH Dicker RC, 1991. Epi Info: A general-purpose microcomputer program for public health information systems. Am J Prev Med 7: 178–182. 1657068

[16] Worku H , 2022. Knowledge, attitude, and practice of community towards an onchocerciasis elimination program from south west Ethiopia. J Trop Med 2022: 1–11. 10.1155/2022/1417804 PMC9249543 35784943

[17] Rwakimari JB Hopkins DR Ruiz-Tiben E, 2006. Uganda’s successful Guinea Worm Eradication Program. Am J Trop Med Hyg 75: 3–8. 16837699

